# Aspects of embryo-maternal communication in establishment of pregnancy in cattle

**DOI:** 10.21451/1984-3143-AR2019-0075

**Published:** 2019-10-22

**Authors:** José M. Sánchez, Constantine A. Simintiras, Patrick Lonergan

**Affiliations:** School of Agriculture and Food Science, University College Dublin, Belfield, Dublin 4, Ireland.

**Keywords:** conceptus, fertility, bovine, uterus, progesterone

## Abstract

Establishment of pregnancy in mammals requires reciprocal molecular communication between the conceptus and endometrium that modifies the endometrial transcriptome and uterine luminal milieu to support pregnancy. Due to the small size of the early embryo and elongating conceptus relative to the volume of the uterine lumen, collection of endometrium adjacent to the developing conceptus is difficult following conventional uterine flushing methods in cattle. Use of endometrial explants in culture can overcome this challenge and reveal information about the dialogue between the developing embryo and the uterus. The aim of this short review is to summarize some of our recent findings in relation to embryo maternal interaction during bovine pregnancy establishment and to put them in the wider context of fertility in cattle.

## Introduction

Embryo mortality is a major contributor to poor reproductive efficiency in dairy and beef cows. A significant proportion of embryonic loss in cattle, particularly lactating dairy cows, occurs during the first 2-3 weeks after conception, before maternal recognition of pregnancy, which occurs around Day 16. In a recent comprehensive review, Wiltbank *et al*. (2016) described four pivotal periods of pregnancy loss during the first trimester of gestation and discussed possible causes for pregnancy failure during these periods. Despite a relatively high fertilization rate (>85%), 20%-50% of high-producing lactating dairy cows experience pregnancy loss during the first week of gestation. From Days 8 to 27, concomitant with embryo elongation and maternal recognition of pregnancy, losses average approximately 30%. From Days 28 to 60, losses of approximately 12% occur while in the fourth period, during the third month of pregnancy, pregnancy losses are reduced (approximately 2%), but may be elevated in some cows, particularly in those carrying twins in the same uterine horn (Wiltbank *et al*., 2016).

Communication between the developing embryo and the mother is vital for the successful establishment and maintenance of pregnancy but the requirement for this dialogue is temporal in nature and in reality only becomes absolutely essential from around Day 15-16 onwards. Pregnancies are routinely established in commercial embryo transfer following the transfer of 6-7 day old embryos, often produced in vitro, to synchronized recipients. Indeed, pregnancy can be established following the transfer of elongated conceptuses up to at least Day 16 (Betteridge *et al*., 1980; Kimura and Matsuyama 2014) indicating that the uterus does not need to receive any signals from the embryo prior to that stage in order for pregnancy occur.

Up to the blastocyst stage (Day 7-8), the bovine embryo is relatively autonomous, as blastocysts can be produced in vitro in the absence of contact with the female reproductive tract and are capable of establishing pregnancy after transfer to a synchronous uterus. In contrast to primates and rodents, in which implantation occurs shortly after the blastocyst enters the uterus, in ungulates, such as ruminants and pigs, the early conceptus undergoes a phase of rapid growth and elongation before implantation, the latter occurring 2-3 weeks after fertilization. In cattle, conceptus elongation is initiated around Day 13 of gestation when the hatched bovine blastocyst transitions sequentially from a spherical- to ovoid-, then tubular- and finally filamentous-shaped structure that primarily involves proliferation of the conceptus trophectoderm cells. During this time, the elongating conceptus secretes interferon tau (IFNT), the maternal pregnancy recognition signal in ruminants (Bazer and Thatcher 2017). While there is a strong positive correlation between conceptus length and IFNT secretion (Rizos *et al*., 2012), surprisingly, threshold concentrations of IFNT required to overcome luteolysis are as yet not known (Forde and Lonergan 2017; Spencer *et al*., 2017).

Elongation is necessary to ensure sufficient concentrations of IFNT are secreted and to expand the conceptus surface area for maximal vascular exchange with maternal tissues after implantation. An inability of the conceptus to optimally elongate undoubtedly results in embryonic loss and is believed to significantly contribute to reproductive failure in cattle (Wiltbank *et al*., 2016; Moraes *et al*., 2018). In contrast to pre-hatching development, elongation is predominantly maternally-driven, dependent on substances in the uterine lumen fluid (ULF; or histotroph). As evidence for this, blastocysts do not elongate in vitro (Brandão *et al*., 2004) and the absence of uterine glands in vivo results in failure of blastocysts to elongate following embryo transfer (Gray *et al*., 2002).

Spatial and temporal changes of the endometrial transcriptome and histotroph composition are necessary to establish uterine receptivity to implantation and, in turn, are pivotal to the likelihood of successful pregnancy in cattle. Those modifications areprimarily regulated by the steroid hormone progesterone (P4) derived from the corpus luteum (CL) which acts via the endometrium to promote conceptus growth and implantation, as well as conceptus-derived IFNT, which prevents development of the endometrial luteolytic mechanism (Forde and Lonergan 2012; Brooks *et al*., 2014). The role of P4 in uterine receptivity is unequivocal (Lonergan *et al*., 2016; Spencer *et al*., 2016). Low circulating P4 concentrations in the first week after ovulation, as frequently occurs in high-producing lactating dairy cows, are associated with under-developed conceptuses (Forde *et al*., 2012) with an altered transcriptomic signature (Barnwell *et al*., 2016) and a low likelihood of establishing pregnancy (Wiltbank *et al*., 2016). On the other hand, elevated concentrations of circulating P4 in the period immediately after conception have been associated with advanced conceptus elongation (Carter *et al*., 2008), increased IFNT production (Mann and Lamming 2001), and greater pregnancy rates in cattle and sheep (Ashworth *et al*., 1989; Stronge *et al*., 2005; McNeill *et al*., 2006). Despite the definitive association between P4 and conceptus elongation, significant natural variation in age-matched in vivo- (Betteridge *et al*., 1980) and in vitro-derived conceptuses occurs, even amongst conceptuses developing in the same uterus (Clemente *et al*., 2009; Sánchez *et al*., 2019a; b). This would suggest that part of the ability to elongate is intrinsic to the embryo and may be related to oocyte and/or blastocyst quality.

The aim of this short review is to summarize some of our recent findings in relation to embryo maternal interaction during bovine pregnancy establishment and to put them in the wider context of fertility in cattle. Several of the recent studies referred to below have used an endometrial explant co-culture system to elucidate this fine dialogue by examining changes in endometrial gene expression induced by blastocysts (Passaro *et al*., 2018, 2019) or by an elongating conceptus (Mathew *et al*., 2019; Sánchez *et al*., 2019b; Bagés-Arnal *et al*., 2019). Due to the maintenance of normal cellular and extracellular architecture in endometrial explants (Borges *et al*., 2012), some of the limitations of traditional cell culture can be overcome; for example, uterine explants allow the communication between resident populations of endometrial cells which cannot be achieved with current 2D and 3D cell culture technologies.

## Role of progesterone in uterine receptivity and conceptus elongation

Progesterone from the CL induces both temporal and spatial changes in the endometrial transcriptome necessary to establish uterine receptivity, when implantation in the uterus is possible (Forde *et al*., 2009). These changes include down-regulation of the nuclear progesterone receptor (PGR) in the luminal and then glandular epithelium (Okumu *et al*., 2010), which allows expression of genes and secretion of their protein products, as well as active transport of other molecules, required for conceptus elongation.

The capacity of the uterus to stimulate conceptus elongation is primarily dependent on secretions from the luminal and glandular epithelium. However, the timing of conceptus elongation is clearly associated with concentrations of P4 in circulation, which acts via the uterus (Clemente *et al*., 2009) to alter the timing of PGR downregulation and thus onset of expression of key genes required for elongation in cattle (Forde *et al*., 2009) and sheep (Satterfield *et al*., 2006). Consequently, P4 has an indirect effect on the secretion of IFNT by the conceptus, given the strong positive correlation between conceptus length and IFNT production (Rizos *et al*., 2012). In order for P4 output from the CL to be maintained, sufficient quantities of IFNT must be produced by the conceptus by Day 16 to abrogate the luteolytic mechanism and maintain CL function and induce expression of both classical and nonclassical interferon-stimulated genes (ISG) in the different cellular compartments of the endometrium that are proposed to regulate conceptus elongation.

Temporal changes in uterine gene expression occur irrespective of whether the cow is pregnant or not (Forde *et al*., 2009) and it is only during maternal recognition of pregnancy, around Day 16, by which time the conceptus is secreting copious amounts of IFNT (Forde and Lonergan 2017) that major changes in gene expression between cyclic and pregnant endometrium become apparent (Forde *et al*., 2011; Bauersachs *et al*., 2012).

## Blastocyst-induced changes in the endometrium

Pregnancy recognition in cattle is initiated around Day 15-16, both at the physiological and transcriptomic level. Nonetheless, the first week of development is critical as evidenced by the fact that, at least in high-producing dairy cows, about 50% of embryos are no longer viable by Day 6-7 (Sartori *et al*., 2010). Whether communication between the embryo and endometrium at this stage is really important remains to be demonstrated convincingly. There is unequivocal evidence that when development occurs in vivo, blastocyst quality is improved in terms of ultrastructure (Rizos *et al*., 2002a), gene expression profiles (Lonergan *et al*., 2003a, b; Gad *et al*., 2012), cryotolerance (Rizos *et al*., 2002b) and pregnancy rate after transfer (Hasler *et al*., 1995) compared to when blastocysts are produced in vitro. However, evidence of a reciprocal effect of a single embryo on the cells of the uterus is more difficult to detect. As mentioned earlier, the fact that blastocysts can be produced routinely in vitro in the absence of contact with the reproductive tract and subsequently establish a pregnancy after transfer to a recipient supports the notion that exposure of the reproductive tract to the early embryo, or vice-versa, is not required for pregnancy.

In vitro studies have demonstrated that preimplantation embryos secrete a variety of biochemical messengers, embryotropins, that act in an autocrine manner to promote embryonic development (reviewed by Wydooghe *et al*., 2015). For many of these factors, expression of corresponding receptors inthe uterus has been identified, the activation of which could lead to cellular and tissue responses in regions that are in close physical contact with the embryo. Others have reported that the early bovine embryo (from Day 5 to Day 9) induces an anti-inflammatory response in uterine epithelial cells and immune cells in vitro (Talukder *et al*., 2017). Therefore, if factors secreted by the pre-elongating embryo enhance changes in the transcriptome and in the proteome of the endometrium, those changes are most likely to be local in nature and may not be detectable using crude methods of sample collection. Use of an explant model allows the interrogation of cells that were in direct contact with the embryo(s) facilitating the detection of such local embryo-induced changes in the endometrium during the very early stages of pregnancy.

Recently, local embryo-induced alterations in the endometrial transcriptome from spatially-defined regions in response to the presence of a Day 7 bovine embryo were reported (Sponchiado *et al*., 2017). In that study, the presence of an embryo altered the abundance of 12 transcripts in the cranial part of the uterine horn ipsilateral to the CL, including classical ISG (*ISG15, MX1, MX2, OAS1Y*), genes involved in prostaglandin biosynthesis (*PTGES, HPGD, AKR1L4*), water channels (*AQP4*) and a solute transporter (*SLC1A4*); however, the extent of change was relatively minor in nature ranging from 1.35- to 2-fold). Based on this, we hypothesized that the blastocyst induces local changes in the endometrial transcriptome through the production of IFNT and potentially other diffusible factors. Using co-culture of endometrial explants in the absence or presence of blastocysts or medium conditioned by blastocysts, we demonstrated that bovine endometrium responds to the presence of 8-day old blastocysts by upregulating expression of classical ISG (Passaro *et al*., 2018). This effect was (i) specific to the blastocyst stage - earlier stages did not induce gene expression changes, (ii) dependent on the number of blastocysts present - a minimum of 5 blastocysts were required to detect such changes, and (iii) independent of direct contact - the effect was induced by embryos co-cultured on endometrial explants using a cell culture insert (preventing direct contact) as well as by blastocyst-conditioned medium (Passaro *et al*., 2018). While others have reported differential expression of a small number of other transcripts in the endometrium in vivo, induced by the presence of a single blastocyst (Sponchiado *et al*., 2017), or in cultured endometrial cells (Talukder *et al*., 2017; Gómez *et al*., 2018), we failed to detect in endometrial explants using qPCR (Passaro *et al*., 2018).

**Figure 1:**
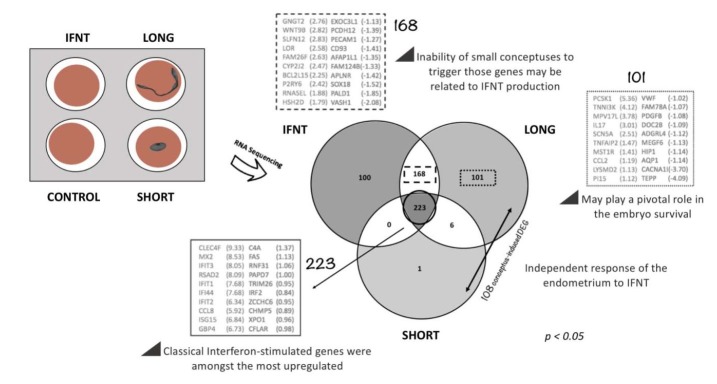
Use of an *ex-vivo* uterine endometrial explant-conceptus co-culture system to elucidate conceptus-induced effects on the endometrium both dependent and independent of interferon-tau (IFNT). Uterine explants taken from the same uterus were exposed to (i) medium alone (control), (ii) 100 ng/ml recombinant ovine IFNT, (iii) a long Day 15 conceptus, or (iv) a short Day 15 conceptus. Numbers of differentially expressed genes indicated for each group are relative to the Control. Modified from Sánchez *et al*. (2019b).

To extend these findings, Passaro *et al*. (2019) used RNA sequencing to investigate global changes in the transcriptome of endometrial explants induced by exposure to blastocysts. Exposure of bovine endometrium to blastocyst-stage embryos resulted in the upregulation of 40 transcripts in blastocyst-exposed endometrial explants compared to the control. Comparison of this list of differentially expressed genes (DEG) with the common list of genes altered in endometrial explants following culture with 100 ng/ml IFNT or a Day 15 conceptus (from Sánchez *et al*., 2019b; Fig. 1) indicated that all of the DEG induced in the endometrium by blastocyst-stage embryos are IFNT-stimulated, in contrast to Day 15 when a significant number of IFNT-independent genes are induced (Mathew *et al*., 2019; Sánchez *et al*., 2019b – see below).

These results support the concept that the early embryo is capable of communicating with the reproductive tract. The effect on the endometrial transcriptome is dependent on the stage of embryo development and appears to be due solely to IFNT. The functional significance, if any, of such induced changes remains to be fully elucidated given that it is possible to transfer embryos from Day 7 onwards to a uterus that has not previously been exposed to an embryo and achieve normal pregnancy rates.

## Response of the endometrium to in vivo or in vitro derived conceptuses

It has been elegantly shown that the endometrium can act as a ‘sensor’, with its transcriptome reflective of the type and developmental competency of the conceptus present (Bauersachs *et al*., 2009; Mansouri-Attia *et al*., 2009). These studies compared the endometrial responses to bovine conceptuses produced by somatic cell nuclear transfer, in vitro fertilization or artificial insemination (AI) and suggested that placental failure in bovine clone pregnancies may originate from abnormal embryo-maternal communication during the peri-implantation period (Day 18-20).

As stated above, it is generally accepted that blastocysts produced in vitro are inferior in quality to in vivo-derived embryos. This difference is reflected in the fact that in commercial embryo transfer, the majority of in vitro-produced blastocysts are transferred fresh while the majority of in vivo-derived blastocysts are transferred frozen (Viana, 2018).

Mathew *et al*. (2019) compared the transcriptomic response of the endometrium following exposure to IFNT or a conceptus derived from the transfer of an in vivo-derived (superovulation and AI) or in vitro-produced (IVF) blastocyst in order to identify novel transcripts dependent and independent on IFNT, conceptus origin and conceptus sex. IVF- or AI-produced blastocysts were transferred into recipient heifers on Day 7 of the estrous cycle. On Day 15, IVF- or AI-derived conceptuses were obtained by uterine flushing and individually placed on endometrial explants in media for 6 h. Explants were also cultured with media alone as a control or media containing 100 ng/mL recombinant ovine IFNT. Incubation of endometrium with IFNT or IVF- or AI-derived conceptuses altered the expression of 491, 498 and 576 transcripts, respectively, compared to the control. Further, 369 DEG were common between explants exposed to IFNT or a conceptus. 240 DEG were uniquely altered by conceptuses (IVF- and AI-derived) but not IFNT. Of these transcripts, 46 were shared between the IVF and AI groups, while 61 and 133 were specific to IVF and AI conceptuses, respectively. Five genes (*MLPH, PROM2, MYADM, VN1R4L, HTR1A*) were more abundant in endometrium exposed to female compared to male conceptuses while a single gene (*ARL4C*) was more abundant in response to male conceptuses than female conceptuses.

These data support the hypothesis that conceptus regulation of gene expression in the endometrium is complex and involves factors other than IFNT that may have a biological role in pregnancy establishment. The findings are consistent with the presence of unique proteins in ULF of pregnant heifers on Day 16 and produced by short-term in vitro cultured Day 16 conceptuses (Forde *et al*., 2015) and those of Bartol *et al*. (1985) who demonstrated that the fully elongated bovine conceptus produces a significant number of proteins when cultured in vitro. Further, Spencer *et al*. (2013) demonstrated that the bovine conceptus produces prostaglandins, which can modify the endometrium prior to pregnancy recognition.

## Effect of conceptus length on the endometrial response

Significant variation in the length and morphology of age-matched conceptuses exists, even when multiple conceptuses are recovered from the same uterine environment (Clemente *et al*., 2009; O’Hara *et al*., 2014), despite the fact that embryos were produced in vitro under the same conditions until the blastocyst stage and were of similar morphological quality at the time of transfer on Day 7. Conceptus length on a given day in the period around pregnancy recognition is thought to be indicative of its quality and the likelihood of establishing and maintaining a pregnancy (Barnwell *et al*., 2016), although this has yet to be definitively established. While significant differences in the transcriptomes of long and short Day 15 conceptuses have been reported (Barnwell *et al*., 2016), the interaction between such divergent conceptuses and the endometrium had, until recently, not been described. We hypothesized that bovine endometrium exposed to long vs. short Day 15 conceptuses would exhibit a different transcriptome profile reflective of potential for successful pregnancy establishment. To test this hypothesis we used a combination of in vitro production of bovine blastocysts, multiple embryo transfer and conceptus-endometrial explant co-culture to investigate the response of the endometrium to age-matched conceptuses of different sizes collected from the same uterine environment (Sánchez *et al*., 2019b). The main findings were that: (i) Day 15 conceptuses vary significantly in length, even when derived from the same uterine environment; and (ii) the endometrium responds in an IFNT-dependent and independent manner to conceptuses of different sizes which likely reflects the ability to successfully establish pregnancy (Fig. 1). These data complement nicely the data on the conceptus transcriptome mentioned above describing differential patterns of mRNA expression between short (mean length of 4.2 ± 0.1mm) and long (24.7 ± 1.9 mm) bovine conceptuses recovered on Day 15 of gestation (Barnwell *et al*., 2016). In that study, a total of 348 genes were differentially expressed related to metabolism and biosynthesis. These genes and cellular pathways involved in enhanced conceptus elongation, as well as the endometrial response blueprint to short and long conceptuses (Sánchez *et al*., 2019b), may ultimately serve as markers of successful pregnancy.

Whether or not smaller conceptuses on a given day are actually abnormal or whether they are simply slower in development is unclear; however, it is likely that they are compromised compared to longer (normal) conceptuses. Definitive proof will come from the recovery and retransfer of long and short age-matched conceptuses to establish their ability to initiate and maintain pregnancy.

While the differences in conceptus length are due, at least in part, to intrinsic differences in the embryo/conceptus, likely related to oocyte quality, it would be wrong to completely discount a role for the uterus in contributing to variation in conceptus length and pregnancy establishment. Using a model of repeated embryo transfer originally described by McMillan *et al*. (1999), Geary *et al*. (2016) classified heifers based on pregnancy success following serial embryo transfer as high fertile (HF), subfertile (SF), or infertile (IF). Conceptus survival and growth to Day 14 was not compromised in SF and IF heifers. However, pregnancy rate on Day 28 was higher in HF (70.4%) than in heifers with low fertility (36.8%; SF and IF). In a follow-up study (Moraes *et al*., 2018), pregnancy rate on Day 17 was substantially higher in HF (71%) and SF (90%) than IF (20%) heifers. Furthermore, elongating conceptuses were about twofold longer in HF than SF heifers. Taken together, these data suggest that the uterus impacts conceptus survival and programs conceptus development, and effects of dysregulated conceptus-endometrial interactions elicit loss of the post-elongation conceptus in SF cattle during the implantation period of pregnancy.

In summary, bovine endometrium responds differently in terms of its gene expression signature to age-matched long and short conceptuses, in an IFNT-dependent and independent manner, which may be critical for embryo survival. In particular, short conceptuses failed to alter the expression of a large number of ISG that were altered by both IFNT and long conceptuses, suggesting that insufficient IFNT production is a major contributory factor to lower survival of such conceptuses. Furthermore, the alteration of >100 endometrial transcripts uniquely by long conceptuses suggests that other aspects of maternal-embryo communication at this critical time are IFNT-independent.

## Differential response of endometrium ipsilateral and contralateral to the CL

Embryo transfer studies established that the incidence of embryo loss is higher following transfer to the uterine horn contralateral to the ovary containing the CL compared to transfer to the ipsilateral horn (Christie *et al*., 1979). Whether these differences are manifest in conceptus growth and elongation in the critical window preceding maternal recognition of pregnancy is unknown. Knowledge of differences in gene expression between the uterine horns during the estrous cycle could further enhance our understanding of uterine receptivity and the process of conceptus elongation, key events for the maternal recognition of pregnancy and, in turn, successful pregnancy establishment.

We hypothesized that differences in the endometrial transcriptome between the ipsilateral and contralateral horns throughout the cycle exist, and those differences would be correlated with differences in conceptus elongation after embryo transfer (Sánchez *et al*., 2019a). Endometrial samples from both horns were collected from synchronized heifers slaughtered on Day 5, 7, 13 or 16 post-estrus and subjected to RNA sequencing. Main findings were that: (i) day of the estrous cycle contributed to the greatest variation in the endometrial transcriptome; (ii) there were many more altered genes between the uterine horns ipsilateral and contralateral to the CL in the early (Day 5 and 7) as compared to late (Day 13 and 16) luteal phase; (iii) signalling pathways regulating pluripotency of stem cells were highly dysregulated when both uterine horns were compared, regardless of the day of luteal phase. In a separate experiment within the same study, ten Day 7 in vitro produced blastocysts were transferred into the uterine horn ipsilateral or contralateral to the CL or into both horns (i.e., bilateral) of synchronized recipient heifers. Reproductive tracts were recovered at slaughter on Day 14 and the number and dimensions of recovered conceptuses were recorded for each horn. Site of embryo transfer did not affect recovery rate (48.0%, 168/350) or length of conceptuses. Thus, although differences in gene expression exist between the endometrium of uterine horns ipsilateral and contralateral to the CL in cattle, these differences were not associated with a reduced ability of the uterus to support conceptus survival or development to Day 14 after embryo transfer on Day 7.

In a follow-on study, we asked whether the endometrium from the uterine horn ipsilateral or contralateral to the CL responds differently to an elongating conceptus. Bagés-Arnal *et al*. (2019) compared the local response of the ipsilateral and contralateral endometrium to a Day 14 conceptus. Although no differences in gene expression were detected between ipsilateral and contralateral endometrium, the response of the endometrium to a Day 14 conceptus was distinct in each uterine horn. Interestingly, more genes were differentially expressed in the contralateral than in the ipsilateral endometrium after exposure to a conceptus 239 vs. 61 DEG, respectively). Many of the biological processes enriched in the DEG between both horns in response to a conceptus were associated with immune response and response to stimuli. This observation is consistent with the study of Moraes *et al*. (2018), where relatively few differences were detected in theendometrial transcriptome of non-pregnant high-fertile, subfertile and infertile heifers; however, the response of the endometrium from high-fertile and subfertile animals to pregnancy was remarkably different (3422 vs. 1095 DEG, respectively).

These data extend those of Sánchez *et al*. (2019a) describing temporal changes in the transcriptome of the endometrium ipsilateral and contralateral to the CL during a nonpregnant estrous cycle by describing differential response of the endometrium in both uterine horns to an elongating conceptus. The large difference in the number of DEG between the endometrium ipsilateral and contralateral to the CL in response to a Day 14 conceptus may be related to the differences in P4 concentrations during the first days after ovulation (Takahashi *et al*., 2016), since, as mentioned earlier, P4 is one the major regulators of the uterine receptivity through changes in the endometrium transcriptome.

## Uterine lumen fluid compositon

The composition of ULF during the preimplantation period has been extensively studied in sheep (see review by Bazer *et al*., 2015 and references therein). Data in cattle are more limited although various studies have reported on aspects of ULF composition under various physiological states (Mullen *et al*., 2012; Faulkner *et al*., 2013; Forde *et al*., 2015).

We recently metabolically interrogated ULF flushes on Days 12-14 - the window of conceptus elongation-initiation - from cyclic heifers, either (i) supplemented with P4 on Day 3 post-estrous (high P4 cohort), or (ii) not (normal P4 cohort; physiological control). The former group is an established model of conceptus elongation rate acceleration (Carter *et al*., 2008; Clemente *et al*., 2009; O’Hara *et al*., 2014). Given that conceptus elongation coincides with a period of significant bovine pregnancy loss, our aim was to achieve a better understanding of the biochemical landscape surrounding the peri-elongation conceptus. Over 5000 metabolites were screened for by high-throughput untargeted ultra-high-performance liquid chromatography tandem mass spectroscopy, with 233 consistently identified, clustering within 8 super-pathways: amino acids, carbohydrates (Simintiras *et al*., 2019a), lipids (Simintiras *et al*., 2019b), cofactors, vitamins, nucleotides, peptides, energy substrates, and xenobiotics (Simintiras *et al*., 2019c). A global analysis of this dataset revealed three core ‘strategies’ likely utilised by the bovine endometrium to facilitate conceptus elongation, discussed below.

Firstly, indicative of the changing biochemical requirements of the conceptus around the initiation of elongation, a metabolic shift in the ULF of normal P4 heifers after Day 12 was observed (Simintiras *et al*., 2019d), to which fructose and mannitol/sorbitol were central. More specifically, only these two metabolites increased on Days 13 and 14 vs. 12 within the normal P4 group. Moreover, fructose and mannitol/sorbitol were elevated by 18.4 and 28.4-fold, respectively, in the ULF of high vs. normal P4 heifers on Day 12 (Simintiras *et al*., 2019a) - the greatest differences observed throughout the study - suggestive of a key role for these metabolites in sustaining, in addition to initiating, conceptus elongation.

Secondly, sub-pathway enrichment and representation analyses revealed that metabolic cascades of likely importance to conceptus elongation-initiation revolve around phospholipids, polyamines, and purines. Regarding the former, membrane biogenesis is intuitively essential to the ~30-fold increase in trophoblast length between Days 12-15 (Betteridge *et al*., 1980; Brooks *et al*., 2014). As 47% of identified lipids were intricately linked to membrane biogenesis, it seems reasonable to suspect that endometrial lipid secretions contribute to conceptus membrane fusion, and, thus, elongating conceptus membrane biogenesis is not entirely de novo (Simintiras *et al*., 2019b). The latter, polyamines and purines, are discussed below within the context of adenosine monophosphate signaling.

Thirdly, P4 supplementation amplified the total mean metabolite abundance on Day 14 (P ≤ 0.0001); however, just 19 metabolites (8.2% of total) were elevated (P ≤ 0.05) on Day 14 in high vs. normal P4 heifers, and are, therefore, largely responsible for raising the mean (Simintiras *et al*., 2019d). The biochemical properties of the these ‘selectively’ amplified 19 metabolites - including glucose (primary fuel source), trimethylamine-N-oxide (protein-stabilizing osmolyte), and phenol sulfate (relatively metabolically inert yet acidic molecule) - (i) support the notion that optimal conceptus elongation is contingent on biophysical and physicochemical, in addition to metabolic, cues, and (ii) contribute to the generation of our hypothesis pertinent to the molecular bases of conceptus elongation initiation, discussed below.

**Figure 2:**
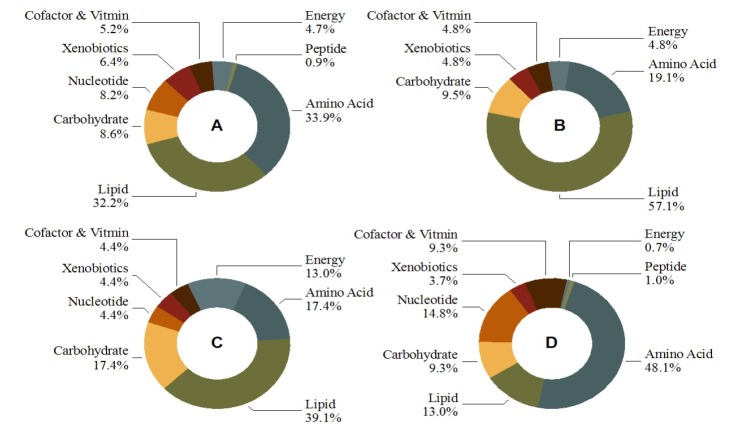
Breakdown of metabolites identified in uterine luminal fluid, by super-pathway, on Days 12-14 of cyclic heifers in terms of total: (A) abundance, (B) day effects, (C) progesterone (P4) effects, and (D) day by P4 interactions. Adapted from Simintiras *et al*. (2019d).

These findings combined, coupled with previous data on the enzymatic profile of bovine ULF (Muñoz *et al*., 2012; Forde *et al*., 2014), give rise to the hypothesis that conceptus elongation internally hinges on 5' adenosine monophosphate-activated protein kinase (AMPK) and peroxisome proliferator-activated receptor gamma (PPARγ) activity, and is modulated by glucose, adenine, and adenosine mono- (AMP), di- (ADP), and tri-phosphate (AMP) influx (discussed in Simintiras *et al*., 2019d). Additional observations worth highlighting include: (i) that total ULF metabolite abundance (Fig. 2A) is not indicative of activity in terms of total day effects (Fig. 2B), P4 effects (Fig. 2C), or day by P4 interactions (Fig. 2D), and (ii) the identification of a plethora of microbiome-associated molecules in ULF, some of which were responsive to P4 (Simintiras *et al*., 2019c), highlights a need for further research into the influence of the uterine microbiome in uterine metabolism and maternal-embryo communication.

## Conclusion

The period of early embryo development and pregnancy establishment is fascinating. This complex process encompasses ovulation, fertilization, blastocyst formation and growth into an elongated conceptus,pregnancy recognition signalling, and development of the embryo and placenta. Despite the aforementioned advances in the field, there is still much to learn. The precise drivers of conceptus elongation remain unknown. While the process is dependent on the uterus - it does not occur in vitro - there is significant variation exhibited amongst conceptuses which is independent of the uterus and may point to variation in oocyte and early embryo quality. Furthermore, the role of the sire in determining embryo quality and in conceptus development is only beginning to be appreciated (Ortega *et al*., 2018).
